# Comparison of surgical invasiveness, hidden blood loss, and clinical outcome between unilateral biportal endoscopic and minimally invasive transforaminal lumbar interbody fusion for lumbar degenerative disease: a retrospective cohort study

**DOI:** 10.1186/s12891-023-06374-1

**Published:** 2023-04-10

**Authors:** Xinle Huang, Wenkai Wang, Guangxing Chen, Xiangchen Guan, Yue Zhou, Yu Tang

**Affiliations:** 1 Department of Orthopaedics, The Second Affiliated Xinqiao Hospital of Army Medical University, Chongqing, China; 2 Center for Joint Surgery, Southwest Hospital, Army Medical University, Chongqing, China; 3 Department of Orthopedics, The Second Affiliated Hospital of Chongqing Medical University, Chongqing, China

**Keywords:** Unilateral biportal endoscopy, Lumbar interbody fusion, Lumbar degenerative diseases, Hidden blood loss

## Abstract

**Background:**

Currently, hidden blood loss (HBL) has been paid more and more attention by spine surgeons. Simultaneously, it has been the effort of spine surgeons to explore more advantages of minimally invasive surgery. More and more articles have compared unilateral biportal endoscopic lumbar interbody fusion (BE-LIF) and minimally invasive transforaminal lumbar interbody fusion (MIS-TLIF). But so far, there is no HBL comparison between BE-LIF and MIS-TLIF. This study aims to compare the surgical invasiveness, hidden blood loss, and clinical outcome of BE-LIF and MIS-TLIF and to provide insight regarding minimally invasive surgery for lumbar degenerative disease (LDD).

**Methods:**

We enrolled 103 eligible patients with LDD who underwent BE-LIF (n = 46) and MIS-TLIF (n = 57) during August 2020–March 2021. We collected data, including demographics, perioperative haematocrit, operative and postoperative hospital times, serum creatine kinase (CK) and C-reactive protein (CRP) levels, and hospitalization costs. Total and hidden blood loss was calculated. Clinical outcomes were assessed using a visual analogue scale (VAS) score for back and leg pain, Oswestry Disability Index (ODI), modified MacNab criteria, fusion rate, and complications.

**Results:**

Basic demographics and surgical data were comparable. The CRP and CK levels were generally lower in the BE-LIF than in the MIS-TLIF group, especially CRP levels on postoperative day (POD) three and CK levels on POD one. True total blood loss, postoperative blood loss, and hidden blood loss were significantly reduced in the BE-LIF group compared with the MIS-TLIF group. Postoperative hospital times was statistically significantly shorter in the BE-LIF group. The VAS pain and ODI scores improved in both groups. At three days and one month, the VAS lower back pain scores were significantly better after BE-LIF. Clinical outcomes did not otherwise differ between groups.

**Conclusions:**

Compared with MIS-TLIF, BE-LIF has similar medium and short-term clinical outcomes. However, it is better regarding surgical trauma, early lower back pain, total and hidden blood loss, and recovery time. BE-LIF is an adequate option for selected LDD.

## Introduction

Minimally invasive transforaminal lumbar interbody fusion (MIS-TLIF) is commonly used to treat lumbar diseases, including spinal stenosis and spondylolisthesis [1]. Compared with open procedures, MIS-TLIF establishes a channel through the paravertebral muscle space, avoids excessive muscle and ligament damage, reduces bleeding and pain, accelerates recovery, and shortens hospitalisation [2]. However, the tubular retractor not only limits the visual field and manoeuvring space but also causes muscle ischemia from extended traction, leading to the occurrence of related complications [3].

Spinal endoscopic techniques have been successively applied to lumbar intervertebral fusion surgery [4]. Recently, biportal endoscopic transforaminal lumbar interbody fusion (BE-LIF) has attracted attention, since the endoscopic and instrument channels are independent of one another. This allows a wider visual field, and unrestricted instrument use [5]. Previous studies have compared BE-LIF and MIS-TLIF [2, 6], reporting that BE-LIF is a viable alternative to lumbar fusion. BE-LIF combines the advantages of minimally invasive endoscopic techniques and flexible operation of conventional instruments. However, relatively little evidence exists regarding serological markers, including creatine kinase (CK) and C-reactive protein (CRP), as objective indicators assessing surgical invasiveness. Additionally, because BE-LIF uses continuous saline irrigation, direct and accurate calculations of blood loss are difficult. Previous studies [2, 7, 8] have reported estimated intraoperative blood loss and drainage volumes to evaluate total blood loss while ignoring hidden blood loss (HBL), such as tissue extravasation and residual blood in dead space. Many studies [9–11] have reported that HBL significantly increased true total blood loss (TBL), which can lower postoperative haemoglobin levels and aggravate anaemia, yielding complications. To date, HBL has not been reported in comparisons of BE-LIF and MIS-TLIF; thus, blood loss comparisons are likely inadequate. This study evaluated postoperative serological markers, TBL, and outcomes to provide a more comprehensive comparison of the two techniques.

## Materials and methods

### Study design and patients

This retrospective cohort study included 103 (46 BE-LIF and 57 MIS-TLIF) patients with clinically and radiographically diagnosed single-segment lumbar degenerative diseases admitted during August 2020–March 2021. During the study period, 21 (eight BE-TLIF and 13 MIS-TLIF) of 103 patients were excluded for the following reasons: lost to follow-up (two BE-TLIF and three MIS-TLIF), coagulation disorders or anaemia (four BE-TLIF and six MIS-TLIF), and incomplete data (two BE-TLIF and four MIS-TLIF). Therefore, the clinical data for comparative analysis came from 38 BE-TLIF and 44 MIS-TLIF patients.

The inclusion criteria were: age ≥ 40 and ≤ 75 years; lower back and lower limb radiation pain ≥ 5 on the visual analogue scale (VAS); and/or neurogenic intermittent claudication not responding to appropriate conservative treatment for over six months; defined single-segment lumbar degeneration or isthmic spondylolisthesis (below Meyerding grade II), lumbar spinal stenosis with spondylolisthesis or instability or lumbar disc herniation with spinal stenosis; and follow-up over one year with sufficient clinical data.

The exclusion criteria were: anaesthesia contraindicated for poor condition; spondylodiscitis, active infection, fractures, or spondylolisthesis (higher than grade II); previous lumbar procedures; cognitive or psychological unfitness for participation; and haematologic-related diseases.

All procedures were performed by the same surgical team from our hospital. The choice of surgical method is a decision made by the patients and their families after the surgeon fully and thoroughly explains the details, advantages and disadvantages, total cost and social medical insurance policy of the different surgical methods. This study was approved by the Medical Ethics Committee of our institution and was performed according to the Declaration of Helsinki. Written informed consent was obtained from all participants.

**Fig. 1:**
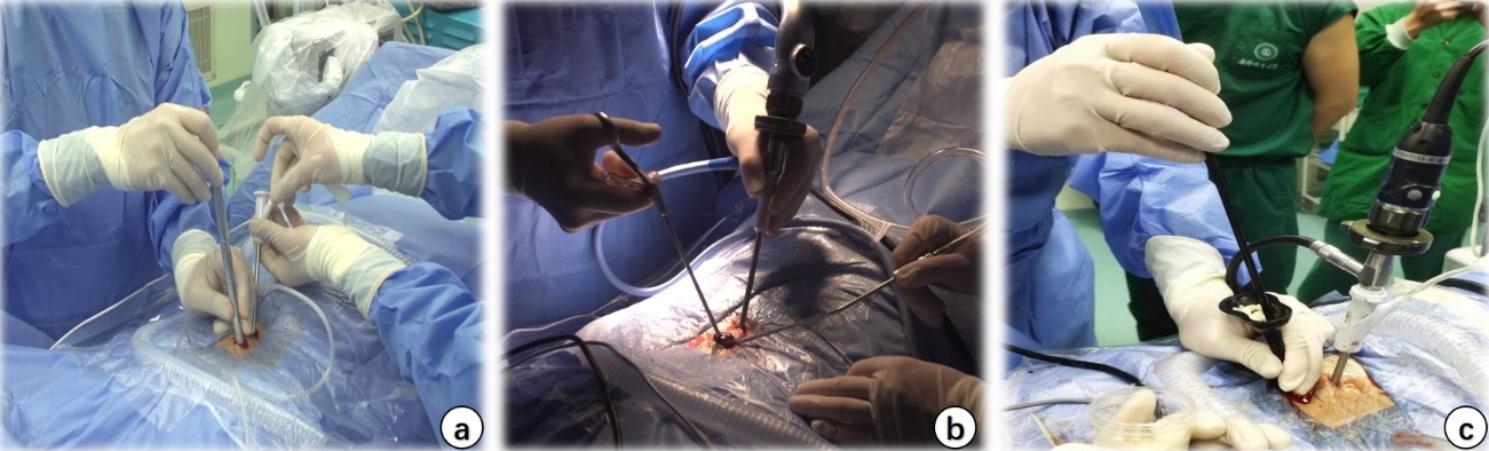
Intraoperative view of BE-LIF. a: Skin dilator used to gradually expand the space and to establish observation and operative channels; b: Intraoperative view; C: Intervertebral bone grafting via an infundibular bone graft device

### Surgical techniques

#### BE-LIF

**Fig. 2:**
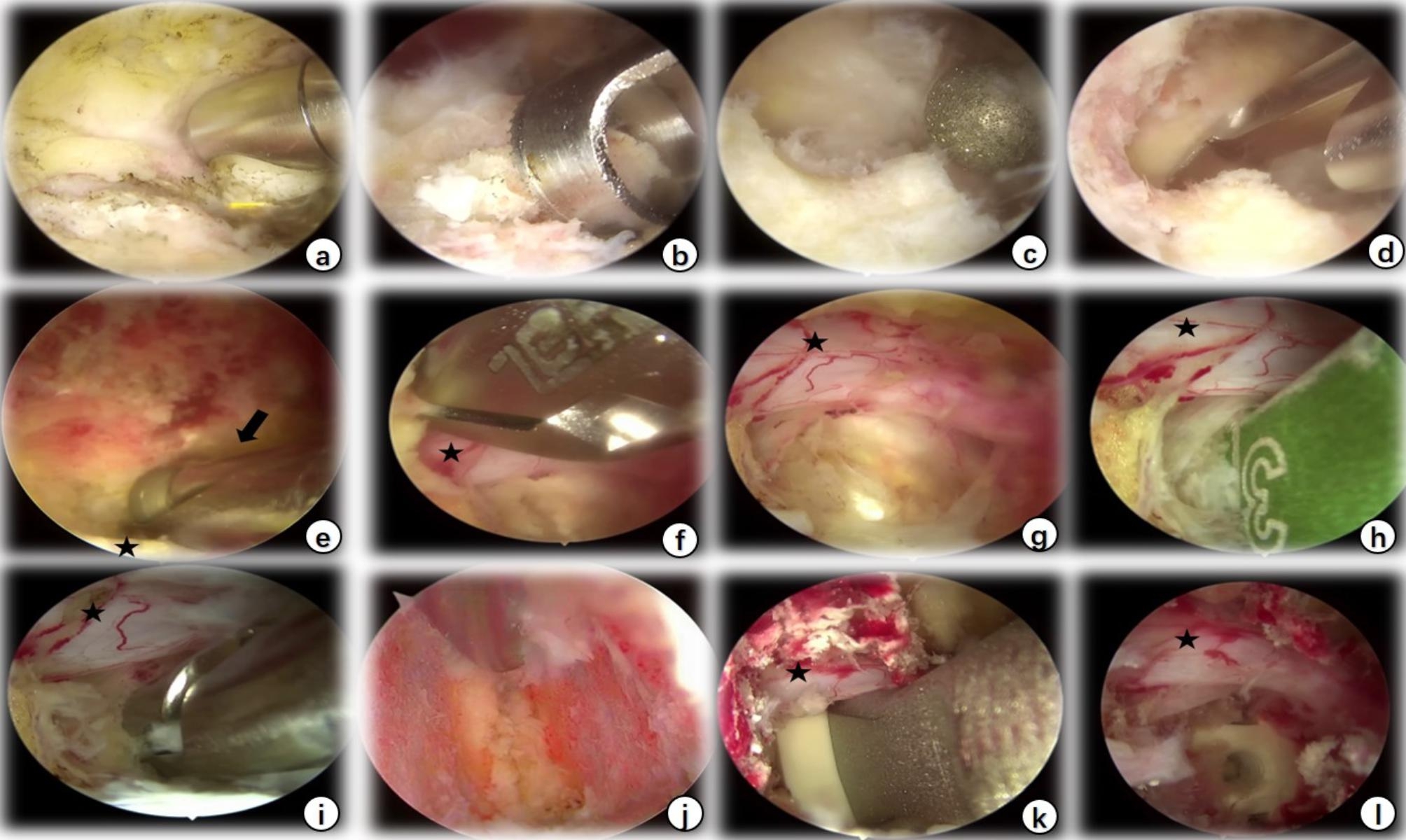
Intraoperative endoscopic view of BE-LIF. The black pentagonal star indicates the dural sac, and the black arrow indicates the contralateral lateral recess. a: The soft tissues on the surface of the lamina and articular process were cleaned with the radiofrequency probe; b, c, d: laminectomy and facet joint resection using a trephine, drill, and Kerrison punch; e: Contralateral lateral recess decompression was performed using a drill; f: Resection of the contralateral ligamentum flavum with the Kerrison punch; g: Exposure of intervertebral space; h, i: Reamer, nucleus pulposus forceps, and other conventional surgical instruments for the management of intervertebral space; j: Cartilage endplate was scraped under the endoscope; k, l: an appropriate size interbody fusion cage was placed under endoscopy

Patients were placed prone after general anaesthesia. The target intervertebral space was identified with G-arm fluoroscopy and marked. After routine disinfection and draping, two 1-cm transverse incisions were made at the medial edge of the upper and lower pedicles in the ipsilateral target space. The soft tissues on the laminar and facet joint surfaces were peeled off with a periosteal stripper, and the skin dilator was used to gradually expand and to establish observation and surgical channels (Fig. 1a and b). Once the light source and irrigation system were connected, the arthroscope was placed within the viewing channel, and the radiofrequency probe was used to clean the soft tissues on the laminar and facet surfaces and to control bleeding to maintain a clear view (Fig. 2a). The lower edge of the ipsilateral upper vertebral lamina and inferior facet joint were resected using a trephine, drill, or Kerrison punch (Fig. 2b, c and d). For patients with lateral recess stenosis, the lateral recess and nerve root canal were decompressed using a high-speed drill and laminar rongeur. For patients with bilateral stenosis, the lamina bone was removed along the spinous process root to the contralateral side to find the contralateral facet joint and lateral recess for contralateral decompression (Fig. 2e). When the ligamentum flavum was exposed, it was removed with a curette, nerve stripper, and Kerrison punch (Fig. 2f); the dural sac and nerve root were sufficiently decompressed to expose the intervertebral space (Fig. 2g). The intervertebral space was then processed with a conventional TLIF instrument reamer, pituitary forceps, and curette (Fig. 2h and i), and the cartilage endplate was curetted under endoscopic direct vision (Fig. 2j). After serial trials, autologous bone, allografts and recombinant human bone morphogenetic protein (rhBMP) were used for interbody bone grafting through an infundibular bone graft device (Fig. 1c), followed by endoscopic placement of an interbody cage (PEEK material, Weigao, Inc., Shandong, China) of appropriate size (Fig. 2k and l). Then, percutaneous pedicle screw fixation was performed using two ipsilateral incisions and two new contralateral incisions. Finally, C-arm fluoroscopy was performed to confirm the final position of the screw, and a drainage tube was placed before suturing the skin.

#### MIS-TLIF

The procedure was performed according to the routine MIS-TLIF technique described in previous reports [12, 13]. After general anesthesia, the patient was placed in a prone position. The target intervertebral space was identified with G-arm fluoroscopy and marked. After routine disinfection and draping, take an 3 cm incision next to the spinous process in the target intervertebral space. Then, the Quadrant retractor system was inserted through the multifidus and Longissimus space. a light source was connected to fully expose the articular process and lamina. Under direct visualization, the Kerrison punch were used to remove part of the upper and lower articular processes and part of the lamina. The ligament flavum was then removed and the nerve root and dural sac were sufficiently decompressed. For patients with bilateral stenosis, contralateral decompression is performed by tilting the working channel. The diseased disc was then removed and the compressed nerve root was released. After careful treatment of the endplate, autologous, allografts and recombinant human bone morphogenetic protein (rhBMP) were used for interbody bone grafting. Then, a standard interbody cage (PEEK material, Weigao, Inc., Shandong, China) were implanted in the intervertebral space. Subsequently, G-arm fluoroscopy confirmed the final location of the cage. Finally, percutaneous pedicle screw fixation was performed and drainage was placed before suturing the wound.

### Postoperative protocol

Both groups followed the same postoperative protocol. Antibiotics were routinely administered. Once the drain was removed according to volume on postoperative day (POD) two or three, patients were encouraged to wear an orthosis to mobilize and to continue protective use for three months. The patients were informed of perioperative precautions and instructed to perform functional rehabilitation. They were instructed to return at one, three, and 12 months after discharge; clinical follow-up was also conducted regularly by telephone or email.

### Demographics, serology, and clinical outcomes

Patients were followed for at least one year. The demographic information of the two groups was recorded preoperatively, including age, gender, body mass index (BMI), diagnosis, and surgical segment. Perioperative data included operative time, perioperative TBL, HBL, and postoperative hospital stay and hospitalization costs. CK and CRP levels were recorded preoperatively and on PODs one, three, and five. Clinical efficacy was evaluated using the VAS and Oswestry Disability Index (ODI) scores of lower back and leg pain preoperatively and three days, one month, three months, and one year postoperatively. The modified MacNab score at one year was used to evaluate patient satisfaction. Perioperative complications were recorded. At one year, radiography and computed tomography (CT) were performed, and fusion rates were assessed by two radiologists according to the Bridwell grading system, with grades I and II defined as spinal fusion.

### TBL and HBL calculations

Haematocrit (HCT) changes can reflect TBL [14]. TBL was calculated according to Gross et al.’s [15] formula: TBL = patient blood volume (PBV)×(HCT_pre_–HCT_post_)/HCT_ave_ (HCT_pre_=preoperative HCT; HCT_post_=HCT on POD three [16, 17], and HCT_ave_=average of HCT_pre_ and HCT_post_). PBV was calculated according to Nadler et al. [18]: PBV = k1×height (m)^3^+k2×weight (kg) + k3 (k1 = 0.3669, k2 = 0.03219, k3 = 0.6041 for men, and k1 = 0.3561, k2 = 0.03308, k3 = 0.1833 for women). HBL was calculated using the method of Sehat et al. [19]. No blood transfusions were performed, thus HBL = TBL– (intraoperative + postoperative blood loss). The volume of blood in the suction bottle was calculated by deducting the irrigation fluid used during the procedure from the volume of liquid in the suction bottle after surgery. The weight of blood infiltrated in the gauzes was calculated by weighing the gauzes before and after surgery and converted it to the volume by dividing density of blood. Intraoperative blood loss was calculated as the sum of blood loss in the suction bottle and soaked gauzes. If a complete blood count was performed while the drain remained, the total amount of blood in the drain collector at that time was recorded.

### Statistical analyses

Statistical software SPSS 27 (IBM Corp., Armonk, NY, USA) was used for data analysis. Measurement data are presented as means ± standard deviations. Repeated-measures analysis of variance (ANOVA) was performed to determine differences in repeated measurements, such as serological markers (CRP and CK) and clinical outcomes (VAS and ODI) between the two groups, and changes in each group over time. Normally distributed variables were assessed using the Student’s T-test, while non-normally distributed variables and ranked data were evaluated using the non-parametric Mann-Whitney U test, such as modified MacNab criteria. Categorical data were analysed with using the chi-square test or Fisher’s exact test. Significance was assessed at P < 0.05.

## Results

### Basic demographics and serology

**Fig. 3:**
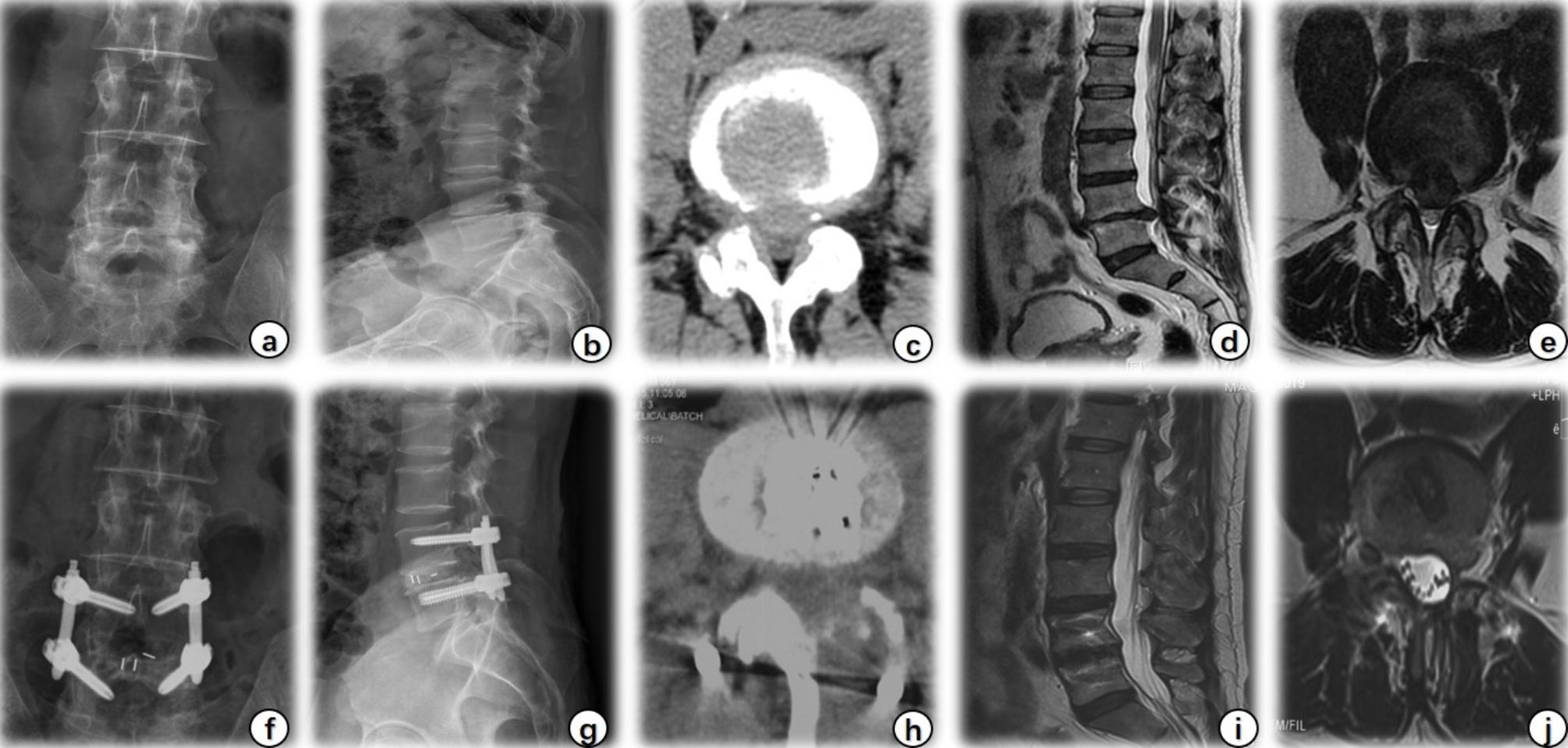
A typical BE-LIF case. A 57-year-old man presented with recurrent lower back pain radiating to the left lower limb with intermittent claudication for six years, which worsened and failed to respond to conservative treatment for six months. Diagnosis: lumbar disc herniation with spinal stenosis. A, B: Preoperative anteroposterior and lateral X-rays of the lumbar spine; c, d, e: Preoperative axial CT, sagittal and axial MRI showed L4/5 lumbar disc herniation with spinal canal stenosis; f, g: Postoperative anteroposterior and lateral X-rays of the lumbar spine; h: Axial CT after BE-LIF showed sufficient spinal canal decompression and local bone defect; i, j: Postoperative sagittal and axial MRI indicated adequate decompression

**Fig. 4:**
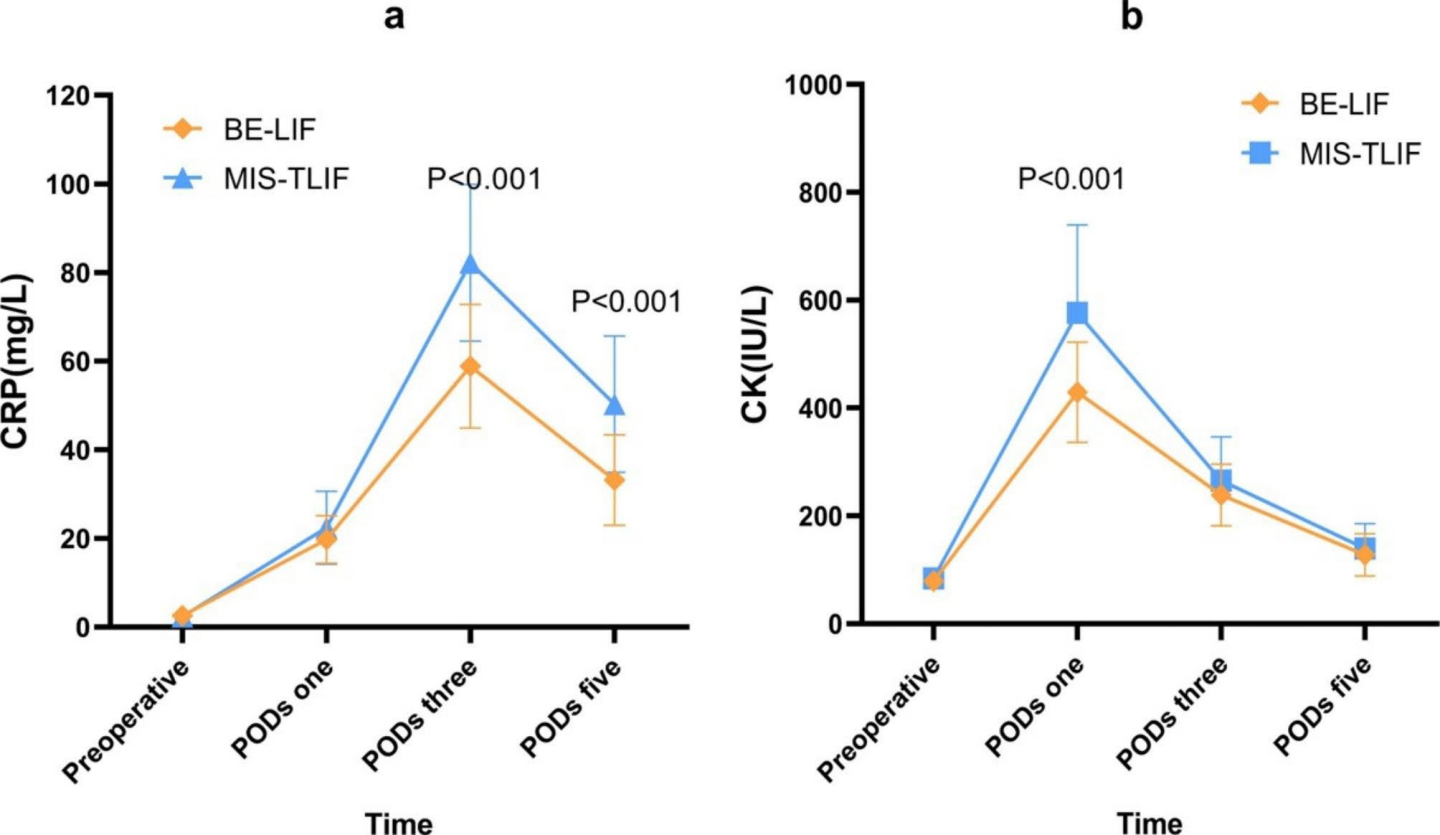
The change trends of (a) C-reactive protein (CRP) and (b) creatine kinase (CK) in the two procedural groups at different time points

Procedures were successful in all patients, and follow-up lasted for more than 12 months. One BE-LIF case is presented in Fig. 3. Preoperative demographics were comparable between the two groups (P < 0.05, Table 1). Preoperative CK and CRP levels did not differ between groups (P = 0.596 and P = 0.196, respectively). Overall, CRP and CK levels were significantly lower in the BE-LIF than in the MIS-TLIF group (P = 0.003 and P = 0.023, respectively). On PODs three and five, CRP level was significantly lower in the BE-LIF group (58.87 ± 13.93 mg/L, 33.12 ± 10.24 mg/L) than in the MIS-TLIF group (82.19 ± 17.69 mg/L, 50.28 ± 15.36 mg/L; P < 0.05 for each; Fig. 4a). Meanwhile, CK levels were significantly higher in the MIS-LIF group (576.26 ± 163.43 IU/L) than in the BE-LIF group (429.36 ± 92.95 IU/L; P < 0.05) on POD one (Fig. 4b).

**Table 1:** Basics Demographics Information and surgical characteristics of the Enrolled Patients

··	Group BE-LIF	Group MIS-TLIF	P Vale
	N=(38)	N=(44)	
Gender (male:female)	22:16	26:18	0.913
Age (years)	60.13 ± 7.36	59.68 ± 6.94	0.778
BMI (kg/m 2)	24.96 ± 1.51	25.10 ± 1.25	0.650
Diagnosis			0.887
Degenerative spondylolisthesis	19	22	
Central stenosis with segmental instability	5	7	
Isthmic spondylolisthesis	4	6	
lumbar disc herniation with spinal stenosis	10	9	
Levels			0.642
L3-4	0	1	
L4-5	28	30	
L5-S1	10	13	
Operation time(mins)	154.23 ± 13.70	131.88 ± 15.02	< 0.001
Hospitalization costs (RMB)	66813.68 ± 3734.79	58968.95 ± 4757.98	< 0.001
Postoperative hospital stays (days)	5.78 ± 0.74	6.72 ± 1.12	< 0.001
Complications rates(%)	5.2%	4.5%	0.489

### Perioperative and clinical outcomes

**Fig. 5:**
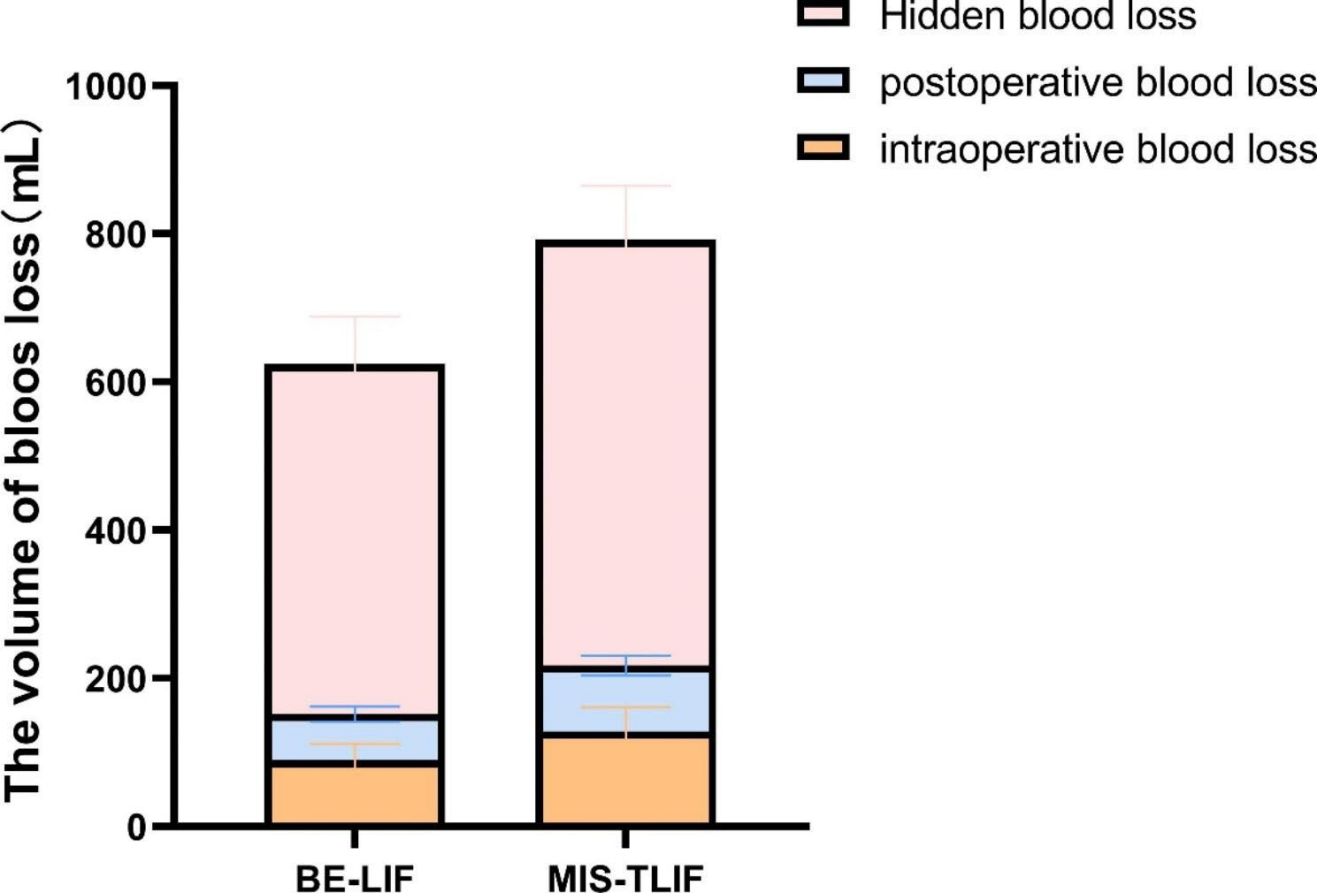
Comparison of blood loss volume of the BE-LIF and MIS-TLIF groups

The mean operative time was longer for the BE-LIF group than for the MIS-TLIF group (154.23 ± 13.70 min vs. 131.88 ± 15.02 min; P < 0.05). Hospitalisation costs were significantly greater for the BE-LIF group (66813.68 ± 3734.79 RMB) than for the MIS-TLIF group (58968.95 ± 4757.98 RMB; P < 0.001). The postoperative hospital stay was significantly shorter for the BE-LIF group (5.78 ± 0.74 days vs. 6.72 ± 1.12 days; P < 0.001; Table 1). Preoperative HCT and PBV were comparable between groups. True TBL, intraoperative blood loss, postoperative blood loss, and HBL were significantly less in the BE-LIF compared with the MIS-TLIF group (Fig. 5). Based on the repeated-measures ANOVA, the preoperative VAS pain and ODI scores did not differ between groups. All pain scores significantly improved with time. No group differences were observed in the leg pain VAS scores at three days and one month or lower back and leg pain VAS or ODI scores at three and twelve months (P > 0.05). However, lower back VAS scores improved more in the BE-LIF group than in the MIS-TLIF group three days and one month after surgery (P < 0.001; Table 2). According to the modified MacNab criteria, at 12 months, the excellent and good rates were 97.3% and 95.4% in the BE-LIF and MIS-TLIF groups, respectively, with no significant difference (P > 0.05; Table 2). Bridwell grading was used for interbody fusion (Table 2). In the BE-LIF group, there were 25 grade I, nine grade II, and four grade III cases; the fusion rate was 94.7%. In the MIS-TLIF group, there were 28 grade I, 11 grade II, and five grade III cases, with a fusion rate of 95.4%. The fusion rates did not differ between the two groups (P > 0.05; Table 2). No major complications occurred in either group. Two small dural tears occurred during BE-LIF. In the MIS-TLIF group, there were two cases of transient ipsilateral dysesthesia All patients recovered with conservative treatment.

**Table 2:** Comparison of Follow-up Outcomes in Group BE-LIF and Group MIS-TLIF

	Group BE-LIF	Group MIS-TLIF		P Value
VAS of low-back pain				<0.001
Preoperative	6.26 ± 0.79	6.13 ± 0.76		0.465
Postoperative 3 day	3.60 ± 1.19*	4.63 ± 0.96*		<0.001
1 months	2.65 ± 0.74*	3.33 ± 0.86*		< 0.001
3 months	2.26 ± 0.55*	2.40 ± 0.54*		0.233
12 months	1.31 ± 0.47*	1.40 ± 0.49*		0.388
VAS of leg pain				0.272
Preoperative	5.84 ± 1.34	6.09 ± 1.62		0.457
Postoperative 3 day	1.78 ± 0.74*	1.65 ± 0.91*		0.484
1 months	1.31 ± 0.70*	1.18 ± 0.65*		0.375
3 months	1.05 ± 0.61*	0.88 ± 0.57*		0.211
12 months	0.63 ± 0.48*	0.77 ± 0.67*		0.289
ODI				0.098
Preoperative	58.10 ± 11.92	55.43 ± 9.19		0.256
Postoperative 3 day	26.63 ± 6.97*	29.04 ± 6.13*		0.099
1 months	18.05 ± 3.70*	19.68 ± 3.97*		0.060
3 months	13.39 ± 4.63*	14.27 ± 3.72*		0.345
12 months	10.42 ± 3.81*	9.95 ± 3.03*		0.539
Fusion rate (grade I, II)†	94.7%	95.4%	1.000	
Grade I	25	28		
Grade II	9	11		
Grade III	4	5		
Grade IV	0	0		
Modified Macnab criteria※	28:9:1:0	30:12:2:0	0.919	
(Excellent and good) rate	97.3%	95.4%	1.000	

*: Compared with preoperative, P < 0.05

VAS: visual analog scale, ODI: Oswestry Disability Index

†: Bridwell interbody fusion grading system: Grade I is defined as a fusion with remodeling and trabeculae present; Grade II is an intact graft with incomplete remodeling and no lucency present; Grade III is an intact graft with potential lucency at the cranial or caudal end; Grade IV is absent fusion with collapse/resorption of the graft

※: excellent: good: fair:poor

## Discussion

In this study, the VAS pain and ODI scores improved significantly in both groups at each time point compared with preoperatively. The clinical results at three months and one year were similar between groups, without significant differences. It appears that both surgical techniques are beneficial for LDD patients, with similar medium and short-term clinical results. However, the low back pain VAS scores at three days and one month were better in BE-LIF than in MIS-LIF patients. This suggests that BE-LIF better reduces lower back pain in the early postoperative period. CRP indicates the level of inflammation and tissue trauma [20]. In the absence of infection, the peak postoperative CRP value is assumed to reflect the extent of tissue damage [21, 22]. CRP levels tend to peak on POD three and decrease rapidly to baseline between PODs ten and 14 [22, 23]. In our study, CRP peaks were similar in the two groups. CRP values were generally lower in BE-LIF than in MIS-TLIF patients and were significantly reduced on POD three. CK is a quantitative indicator of muscle damage, and there is a relationship between CK levels and the pressure exerted on paraspinal muscles by retraction [24, 25]. In a prospective cohort study, Arts et al. [26] observed a dose-response relationship between CK and the degree of surgical invasiveness. In our study, the peak postoperative CK level was measured on POD one in both groups, consistent with previous study results [27]. The CK value was generally lower in the BE-LIF group than in the MIS-TLIF group, and this was more obvious at day one. These results suggest that BE-LIF causes less trauma and muscle damage than MIS-TLIF, which may also explain the different lower back pain scores in the early postoperative period (P < 0.001). In addition, Heemskerk et al. [28] have reported that the effects of pressure and duration of the tubular retractor and retraction of the paraspinal muscles during MIS-LIF will lead to muscle atrophy and denervation, thus increasing the possibility of early postoperative pain. However, the initial BE-LIF technique working area is established within the trigone of the multifidus muscle, the potential gap between the multifidus and spinous process of the posterior lamina [29]. Therefore, BE-LIF preserves the spinal structural integrity as much as possible and reduces intraoperative trauma and bleeding; these are other explanations for reduced early postoperative lower back pain in the BE-LIF group. Hospitalisation was also shorter in the BE-LIF group than in the MIS-TLIF group, implying that the BE-LIF group had better early clinical outcomes.

The concept of HBL was first proposed by Sehat et al. in 2000 [30] and mainly includes tissue extravasation, residual drain blood, and BL caused by haemolysis, which is often ignored by surgeons. Sehat et al. [30, 31] found that after total hip arthroplasty, HBL reached 49% of true TBL. In a prospective analysis of 114 patients, Smorgick et al. [32]reported substantial HBL during posterior spinal fusion surgery. Many studies have shown that ignoring HBL may not only result in postoperative anaemia not matching perioperative blood loss, but also in medical complications including delayed wound healing, infection, and prolonged hospitalisation [10, 17]. Therefore, clarifying HBL allows for a more accurate assessment of TBL. In previous studies [33, 34], HBL ranged from 194.4 to 782.4 ml during MIS-TLIF, consistent with our results. In our study, BE-LIF patients had significantly lower TBL, intraoperative and postoperative blood loss, and HBL than MIS-TLIF patients (Table 3). One possible explanation is that BE-LIF causes less muscle damage than MIS-TLIF and may reduce postoperative haemolysis. Additionally, maintaining adequate saline pressure perfusion and water patency is essential for clear vision under BE-LIF endoscopy. We usually place the saline perfusion solution approximately 70–90 cm above the surgical field surface and maintain pressure at 30–50 mmHg, which is higher than bone and small blood vessel bleeding pressures (approximately 15–25 mmHg). It simultaneously maintains clear vision and reduces bleeding.

**Table 3:** Comparison of TBL and HBL between BE-LIF and MIS-TLIF

	Group BE-LIF	Group MIS-TLIF	P Value
Preoperative HCT (%)	42.10 ± 3.21	41.52 ± 3.09	0.407
patient’s blood volume (PBV) (L)	4.46 ± 0.31	4.35 ± 0.28	0.082
Total blood loss(TBL)(mL)	624.03 ± 80.67	792.01 ± 104.89	< 0.001
intraoperative blood loss(mL)	89.47 ± 21.95	128.52 ± 32.66	< 0.001
postoperative blood loss(mL)	62.36 ± 10.31	88.97 ± 13.31	< 0.001
Hidden blood loss (HBL) (mL)	472.19 ± 64.44	574.51 ± 72.85	< 0.001

HCT: haematocrit

Some studies [2, 35] have reported that continuous saline perfusion flushing of the fusion bed within the intervertebral space during BE-LIF surgery, which leads to decreased blood supply and osteogenic factors, may lead to decreased fusion rates. In our study, however, both groups achieved good fusion rates and did not differ, similar to previous studies [5, 8, 36]. BE-LIF can identify and completely remove cartilage endplates under direct endoscopic vision; this provides a good environment for intervertebral fusion [4, 37]. However, such delicate endoscopic manipulation not only requires surgical experience but may also be time-consuming; this might explain why the BE-LIF group had longer surgical times. In addition, a meta-analysis [7] reported that the learning curve is also a factor, especially in the curve’s early stages, because decompression takes longer. Kim et al. [38] found that with increasing case numbers, the BE-LIF operation time will gradually decrease; proficiency is reached at around 34 cases. Although the operation time was longer in the BE-LIF than in the MIS-TLIF group, the incidence of perioperative complications did not differ. No severe complications requiring revision occurred in either group. According to the modified MacNab criteria, the excellent and good rates were 97.3% in BE-LIF and 95.4% in MIS-TLIF at one-year follow-up. This indicates that both techniques are safe and effective.

We believe that the BE-LIF technique offers several advantages. First, it has independent endoscopic and instrument channels. Therefore, the endoscopic lens has a wide range of movement and flexibility, and identification of intraspinal structures is clearer and more convenient. Second, the space between the muscle and lamina is used to establish the initial working channel, and there is no prolonged paravertebral muscle traction, causing less lower back muscle trauma. Continuous irrigation with water pressure during the operation also helps to reduce bleeding and to maintain a clear view with endoscopy, reducing the infection risk. However, hospitalisation costs were significantly greater in the BE-LIF group (66813.68 ± 3734.79 RMB) than in the MIS-TLIF group (58968.95 ± 4757.98 RMB; P < 0.001). Higher prices and an inadequate Chinese social-medical insurance system related to BE-LIF surgery have somewhat limited its development.

Some limitations of our study should be mentioned. First, this is a single-centre, retrospective cohort study, and inherent selection bias is inevitable. We did our best to balance the two groups at the beginning of the study, and they did not differ based on basic demographics. Second, we calculated true TBL using HCT at three days postoperatively (HCT_post_). If the patient has not yet hemodynamically stabilized, fluid shifts may be incomplete, resulting in low values for calculated TBL and HBL. This factor did not affect the results of our two groups, because the HCT_post_ values of both groups were collected on the same day after surgery. Third, the case number was small and follow-up time short; thus, a prospective randomized controlled trial with longer follow-up and larger sample size is still needed to further evaluate this technique.

## Conclusion

Compared with MIS-TLIF, BE-LIF has similar medium and short-term clinical efficacy and fusion rates. However, BE-LIF is favourable regarding surgical trauma, early postoperative lower back pain, total and hidden BL, and recovery time. These advantages make it an effective option for selected lumbar degenerative diseases. Further studies with large samples and long-term follow-up are needed.

## Data Availability

The datasets used and/or analyzed during the current study are available from the corresponding author on reasonable request.

## Abbreviations

HBL: hidden blood loss
TBL: true total blood loss
BE-LIF: biportal endoscopic transforaminal lumbar interbody fusion
MIS-TLIF: minimally invasive transforaminal lumbar interbody fusion
LDD: lumbar degenerative disease
CK: serum creatine kinase
CRP: C-reactive protein
VAS: visual analogue scale
ODI: Oswestry Disability Index
POD: postoperative day
CT: computed tomography
HCT: Haematocrit
PBV: patient blood volume
HCT_pre_: preoperative HCT
HCT_post_: HCT on POD three
HCT_ave_: average of HCT_pre_ and HCT_post_
